# Application of machine learning approaches to analyse student success for contact learning and emergency remote teaching and learning during the COVID-19 era in speech–language pathology and audiology

**DOI:** 10.4102/sajcd.v69i2.912

**Published:** 2022-08-30

**Authors:** Milka C. Madahana, Katijah Khoza-Shangase, Nomfundo Moroe, Otis Nyandoro, John Ekoru

**Affiliations:** 1 School of Electrical and Information Engineering, Faculty of Engineering and the Built Environment, University of the Witwatersrand, Johannesburg, South Africa; 2 Department of Audiology, School of Human and Community Development, University of the Witwatersrand, Johannesburg, South Africa

**Keywords:** artificial intelligence, audiology, hybrid learning, contact, COVID-19, education, machine learning, emergency remote teaching, speech–language pathology, teaching, blended learning

## Abstract

**Background:**

The onset of the COVID-19 pandemic across the globe resulted in countries taking several measures to curb the spread of the disease. One of the measures taken was the locking down of countries, which entailed restriction of movement both locally and internationally. To ensure continuation of the academic year, emergency remote teaching and learning (ERTL) was launched by several institutions of higher learning in South Africa, where the norm was previously face-to-face or contact teaching and learning. The impact of this change is not known for the speech–language pathology and audiology (SLPA) students. This motivated this study.

**Objectives:**

This study aimed to evaluate the impact of the COVID-19 pandemic on SLPA undergraduate students during face-to-face teaching and learning, ERTL and transitioning towards hybrid teaching and learning.

**Method:**

Using course marks for SLPA undergraduate students, K means clustering and Random Forest classification were used to analyse students’ performance and to detect patterns between students’ performance and the attributes that impact student performance.

**Results:**

Analysis of the data set indicated that funding is one of the main attributes that contributed significantly to students’ performance; thus, it became one of the priority features in 2020 and 2021 during COVID-19.

**Conclusion:**

The clusters of students obtained during the analysis and their attributes can be used in identification of students that are at risk of not completing their studies in the minimum required time and early interventions can be provided to the students.

## Introduction

The coronavirus disease 2019 (COVID-19) pandemic has produced a public health crisis worldwide that has severely affected the economic, health, academic and social fabric of the world community (Khoza-Shangase, Moroe, & Neille, 2021; Maital & Barzani, 2020; McKibbin & Fernando, 2020; Pawar, 2020; Soni, 2020). The spread of COVID-19 has been a catastrophe, and most governments in the world instituted measures to minimise the risks and spread of COVID-19 (Blankenberger & Williams, 2020). Some of the measures taken to curb the spread of the disease were and are not limited to wearing face masks, practising social distancing, isolation for individuals infected or exposed to COVID-19 (WHO, 2021) and varying levels of lockdown where movement is restricted. Most countries, including South Africa, restricted gatherings, movement and also implemented curfews (South African Government, 2021). One of the sectors that was significantly affected by the restrictions on movement was the education sector. COVID-19 significantly reshaped the way education is conducted globally, Dhawan (2020). In South Africa, from the time of colonisation to the decolonisation period, most institutions of higher learning have been dependent on face-to-face teaching and learning (Mgqwashu, 2017; Mpungose, 2020). Face-to-face teaching and learning usually occurs in the presence of an instructor or lecturer who transfers knowledge to students in a designated, demarcated classroom. The instructor or lecturer may use chalkboards, textbooks or any other traditional resource (Mpungose, 2020). In the context of political unrests, student protests, the outbreak of a pandemic or any other emergency situation, the demarcated physical classrooms are not available. In addition to that, face-to-face learning is marked by real-time contact with resources at a specific contact time (Mpungose, 2020; Tularam, 2018). At the early stages of the COVID-19 pandemic, most institutions of higher learning were completely closed in South Africa, as part of national regulations forming the National State of Emergency. To ensure that students completed the academic year successfully, most institutions transitioned to emergency remote teaching and learning (ERTL), which may be defined as a temporary shift of instructional delivery to an alternate delivery mode because of crisis circumstances. Emergency remote teaching and learning involves the use of various remote teaching strategies for education that would under normal circumstances be conducted face-to-face or as blended or as hybrid (Mpungose, 2020). Emergency remote teaching and learning applies aspects of both synchronous teaching –using video conferencing tools, for instance, Zoom, Teams and Google Meets (Bond, Bedenlier, Marín, & Händel, 2021) – and in some instances, utilising the asynchronous type of teaching approach, where students are provided with course material and lecture recordings and given flexibility to study in a self-paced fashion.

Some of the approaches used in ERTL were virtual and digital teaching and learning strategies (Mhlanga & Moloi, 2020). Emergency remote teaching and learning has played a huge role in ensuring that academic institutions in South Africa continue to deliver on their mandates. However, considering the South African history of colonialism and apartheid, which was characterised by racial segregation and injustices, transitioning to ERTL came with its own challenges (Besser, Flett, & Zeigler-Hill, 2020; Khoza-Shangase et al., 2021; McQuirter, 2020; Neuwirth, Jović, & Mukherji, 2021; Rajab, Gazal, & Alkattan, 2020). Thaba-Nkadimene (2020) highlighted that changing from face-to-face learning to ERTL, using digital platforms and currently transitioning towards blended or hybrid teaching, is a change that cannot be easily carried out within a short period of time. This change, which has occurred over the last 24 months, has required institutions of higher learning to intensively invest in digital technologies and academic staff digital training (García-Morales, Garrido-Moreno, & Martín-Rojas, 2021). COVID-19 highlighted and exacerbated the already existing social and economic inequalities in South Africa, and at the beginning of the ERTL, some students did not have the resources required for learning; for example, they lacked the device(s) to access information on digital platforms which were being used as part of the ERTL strategy, and they did not have connectivity access. Thus, the Department of Higher Education and Training collaborated with universities to provide laptops for students (Francis, Valodia, & Webster, 2020; Mpungose, 2020). Some students were faced with the dilemma of exorbitant costs of Internet and connectivity interruption, especially students residing in remote areas (Azionya & Nhedzi, 2021).

To overcome some of the challenges faced because of lack of access and Internet interruption, universities responded by partnering with Internet providers as a mitigating strategy, and a fixed amount of data were made available monthly for students to use in some of the universities (ITWeb, 2020). South African universities also negotiated for zero rating of educational sites, which is a solution that had previously been used during the students’ protests in 2015. Zero rating is where mobile network providers and some Internet service providers do not bill their clients for accessing specific sites, in this context, educational sites. Zero rating means that the academic sites may be accessed without depletion of the student’s data bundle. However, the current reality of education is that pedagogically relevant content is no longer constrained to an institutional website or even to a limited number of websites. In ERTL, students were sometimes required to access audio and visual communication tools, for instance, Microsoft Teams, Zoom and Google Classroom. Zero rating can therefore be perceived as having been narrowly interpreted, and there is a huge disconnect between sites that are zero rated and most sites that are pedagogically relevant to ERTL (Mhlanga, 2020; Tenet, 2021).

Another challenge faced by students in navigating ERTL was frequent power interruptions, which also affect Internet connectivity (Azionya & Nhedzi, 2021; Laher, Bain, Bemath, De Andrade, & Hassem, 2021; Oyedotun, 2020). Power interruption is a well-documented ongoing challenge in South Africa, termed *load-shedding,* and it is currently still being resolved. Students required flexibility to access academic material online and to participate in assessments that were conducted online to accommodate this instability in power supply (Gedala & Connie, 2021; Motsepe Foundation, 2021).

The assessment of students during ERTL was the most challenging aspect, both locally and internationally. The proposed assessment strategies that emerged either supported or disrupted the already existing inequalities that widened access to higher education by previously marginalised individuals (Padayachee & Matimolane, 2021). It is argued by Padayachee and Matimolane (2021) that challenges experienced during ERTL originate from an ingrained perspective of an assessment as a measure of student competence and standard of teaching. The transition to ERTL challenged how assessments have previously been viewed at institutions of higher learning. Re-evaluating teaching and redesigning approaches to assessments seems to have triggered a shift in assessment ideology (Padayachee & Matimolane, 2021). Although these strategies assisted students in coping with the ERTL, both course instructors and students continued to face other challenges in completing the academic year (Padayachee & Matimolane, 2021). In most institutions of higher learning in South Africa, Bachelor of Speech–Language Pathology and Bachelor of Audiology programmes are designed to be completed within a duration of 4 years. The practical or clinical courses may be held at the University’s speech and hearing clinics and at speech and hearing clinics at hospitals, schools, industries and care facilities, within the broader urban and rural context. South African training institutions for Speech–Language and Hearing (SLH) have predominantly applied the traditional and orthodox approach to teaching and learning through in-person contact. Teletraining and telepractice have not been employed extensively (Khoza-Shangase et al., 2021). The COVID-19 outbreak therefore brought to light the need for a paradigm shift in the manner in which clinical training for SLH in South Africa is viewed and conducted (Chin et al., 2021; Khoza-Shangase et al., 2021; Schmutz et al., 2021). In pursuit of solutions during ERTL, application of simulations was presented as one of the ways students can conduct their clinical training (Tabatabai, 2020). In a scoping review by Nagdee, Sebothoma, Madahana, Khoza-Shangase and Moroe (2022) on what has been documented about simulation as a mode of clinical training in health care professions, findings indicate the value and usefulness of simulation but only within a hybrid model of training where it is used in conjunction with traditional approaches to training.

**FIGURE 1:**
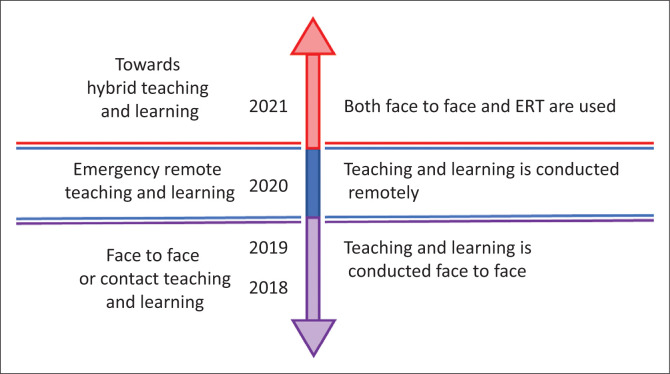
South African academic institution timelines as related to teaching and learning.

Other challenges that were observed during the ERTL were lack of effective peer-to-peer interaction, which is very instrumental in students being able to learn from each other (Chandra & Palvia, 2021). Some students lacked conducive environments at home that would allow for effective ERTL (Gumede & Badriparsad, 2022). These challenges were not restricted to students only, as staff members at institutions of higher learning were also faced with various challenges. Lack of e-tech facilities was one of the setbacks (Mpungose, 2020). Lecturers who were used to only traditional ways of teaching were frustrated with online interactive learning digital technologies because of lack of digital skills or because of the new expectation to have to learn how to use new platforms in a very short period of time, whilst at the same time delivering quality lectures (Engler, 2020). Regardless of all these challenges faced by students and academic members of staff, there was still an expectation that students should excel in their academic work and the academic year be successfully completed. With the ease of the lockdown restrictions, most institutions of higher learning in South Africa are transitioning to hybrid teaching and learning. Hybrid teaching and learning in the current context of most South African universities may be defined as the thoughtful integration of classroom face-to-face learning experiences, ERTL and online learning experiences (Hrastinski, 2019). Figure 1 is a visual representation of the modes of teaching and learning, ranging from face-to-face learning, ERTL and the current transition to hybrid learning; the cohort of students in Figure 1 change each year.

Amidst the COVID-19 pandemic, academic success has remained one of the primary strategic objectives for most institutions of higher learning (Zeineddine, Braendle, & Farah, 2021). With increasing operational costs and budget cuts from government subsidies, companies that sponsor students and general donors terminating their sponsorship contracts and general loss of income by guardians because of COVID-19 (Van Schalkwyk, 2021), academic institutions need to pay more attention to clearly understanding factors that influence students’ performance and be able to address these in their planning and interventions. These interventions would facilitate throughput, thus institutions meeting their mandate. Student retention in light of the COVID-19 pandemic has also been a concern for institutions of higher learning; thus, enrolment and retention of enrolled students has also become a top priority for administrators of institutions of higher learning (Zeineddine et al., 2021). High student dropout rates generally result in lower graduation rates and financial loss for both students and institutions. Institutions that experience high dropout rates may also be ranked lower and their reputation amongst their peers be in disrepute (Cardona, Cudney, Hoerl, & Snyder, 2020). One of the main indicators and predictors of students’ success in academic institutions are the course marks that a student obtains at the end of the course.

In South Africa, most institutions of higher learning use both summative and formative assessments to arrive at course marks for students. The assessments are usually prepared by the lecturer and are moderated by both internal and external stakeholders. The examinations are usually administered by the university, and invigilators are selected by the university. The final course mark is made up of an accumulation of scores over the course period through various tests, practicals and the examination. The final mark is normally presented as a percentage mark, and in many institutions, it is released to the student by the faculty on behalf of the university. A mark of 50% and above in a course would generally be viewed as having passed the course and a mark below that would indicate failure. Course marks are therefore one of the indicators of whether a student has completed a course successfully or not. Institutions of higher learning currently have huge data sets collected over years that can be used to detect and understand unknown patterns and trends and other hidden variable relationships, using data mining techniques and tools.

**FIGURE 2:**
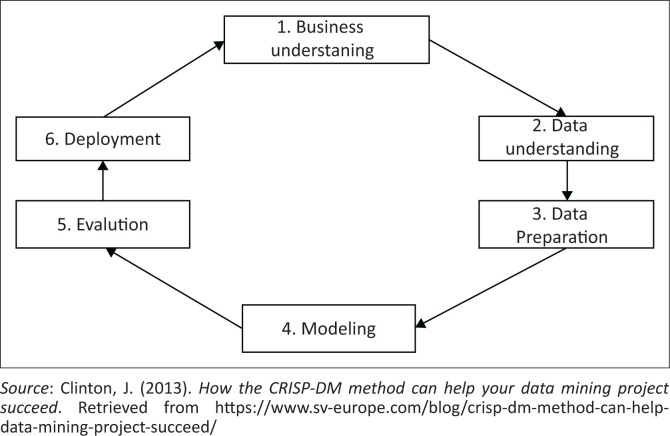
The cross-industry standard process for data mining (CRISP – DM) approach to data mining.

Data mining has in the past been applied in various fields, for instance, health care, business and education. Considering its success in prediction of various critical academic outcomes, performance, retention, success, satisfaction and achievement, data mining may also be applied in assessing the impact of COVID-19 on students’ performance (Alyahyan & Düştegör, 2020). Data mining and application of machine learning (ML) models for classification and analysis of data typically follow a data science approach, namely the Cross Industry Standard Process for Data Mining (CRISP – DM), as shown in Figure 2.

The first step in CRISP-DM is business understanding, which refers to clearly understanding the problem and converting it to a well-defined analytics problem. The current dilemma in this research is to assess the effect of the COVID-19 pandemic on speech–language pathology and audiology (SLPA) undergraduate students under three predefined modes of learning, namely face-to-face teaching and learning, ERTL and transitioning to hybrid teaching and learning. The second stage in this approach entails understanding of the data that are extracted or collected from the data warehouse that are essential in solving the identified problem. Literature shows that prior academic achievements, student demographics, e-learning activities and psychological attributes and environment are the most commonly reported features or attributes that determine students’ success (Alyahyan & Düştegör, 2020). It is reported that 69% of literature articles focused on prior academic achievement and student demographics as a choice of attributes. To benchmark this study, demographic, financial and academic achievements were extracted. The third stage in the CRISP-DM entails data preparation, where the decision of the data to be used for evaluation of the impact of COVID-19 on students’ performance was made. Existing literature (Alyahyan & Düştegör, 2020) was used in determining the criteria for the attributes (columns) and records (rows) that were suitable in answering the research question. The financial, academic and demographic data were merged for easy analysis and interpretation. The fourth stage is the modelling. The two common models used in student success models are predictive and descriptive. To generate patterns that define the basic structure and interconnectedness in students’ data, a descriptive model was firstly used. The most common techniques used are linear regression and logistic regression analysis. Logistic regression is a simple and efficient technique for linear and binary classification models. It is a model that is easy to realise and provides good performance. Logistic regression can be used for binary classification and class probability estimation (Millar, 2011; Tu, 1996). This analysis is commonly used to explain the relationship between a series of predicted variables and a binary variable, for example, whether a student passed or failed. Logistic regression provides the best model that fits the correlation between dependent and independent variable sets (Sinha et al., 2010; Hashim, Awadh, & Hamoud, 2020). Logistic regression is applied for a response variable (*y*) that tracks a binomial distribution (Bielza, Robles, & Larrañaga, 2011). Logistic regression output is defined generically by a probability distribution and has two values: 1 or 0 (Madahana, Ekoru, Mashinini & Nyandoro, 2019).

**FIGURE 3:**
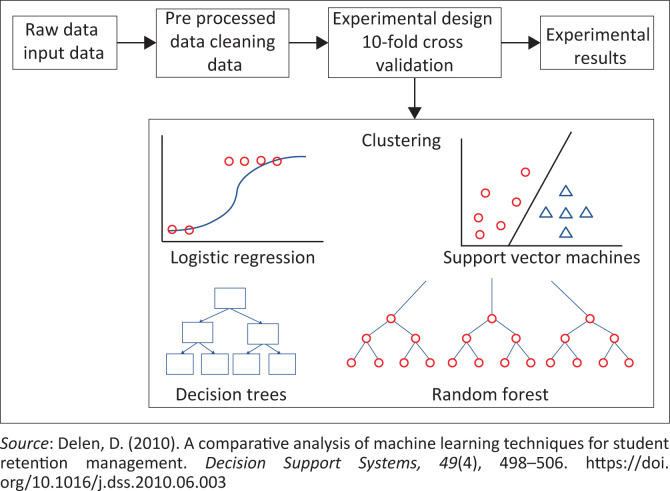
Clustering and classification of students’ data set.

Clustering of students’ data applies to techniques such as K means clustering, which may be defined as one of the statistical and unsupervised analysis that can be performed on a huge data set. This method groups data into homogeneous classes; thus, hidden relationships and patterns can be observed and analysed from the data set. A cluster may be defined as a group of data objects with similar characteristics (Shovon & Haque, 2012). K means clustering is an unsupervised learning algorithm that groups data samples together depending on the attributes, also known as features, that they share. This results in a model that has data samples as inputs and the return groups or clusters that the new data point belongs to. The simplified algorithm is presented by Shovon and Haque (2012). The most commonly used classification algorithms are decision trees and random forest. Decision trees are ML algorithms that apply the branching approach to demonstrate likely outcomes of a decision subject on determined parameters. A hierarchical arranged set of rules makes up the tree structure, which starts with the root attributes and ends with the leaf nodes. One or multiple outcomes from the original data sets represents each tree (Guleria, Thakur, & Sood, 2014; Hamoud, 2017). Decision trees also have root nodes, internal nodes and terminal nodes (Basu, Basu, Buckmire, & Lal, 2019). On the other hand, random forest is a supervised ML algorithm that is constructed from decision trees. The random forest classifier is made up of a combination of tree classifiers, which are generated by using a random vector sampled independently (Breiman, 2001). The other commonly used classification technique is the support vector machine (SVM), which is a ML algorithm that is commonly used for regression and pattern classification (Graf & Wichmann, 2002). The basic operation of SVMs is obtaining an optimal linear hyperplane such that the error of unseen samples is reduced (Chiu & Huang, 2013; Madahana et al., 2019). Figure 3 shows the overall system diagram used for clustering the data sets and for building classification models. The raw data refer to the extracted students’ data, which then undergoes data cleaning, and then classification is conducted. Evaluation is conducted to understand the data mining results. In this study, the patterns and trends observed in students’ performance are analysed. In the evaluation stage, the attributes that were excluded are discussed, as well as whether there is a need to use them in future. The final stage is the deployment where the model can be used.

## Research method and design

### Study design

The research approach selected and applied to this study is a quantitative methodology utilising a correlational study design (Creswell & Creswell, 2017). The design was suitable because correlational studies attempt to establish the extent of a relationship between two or more variables using statistical methods. The chosen approach produces results that can be used in a predictive model, hence its appropriacy for this study.

### Study population and sampling strategy

Data for 605 undergraduate students from the SLPA departments were extracted from the university data warehouse. The data set consists of 95% (*n* = 576) female students and 4.8% (*n* = 29) male students, with age ranges from 18 to 36 years and a mean age of 24.8 in the sample.

### Data collection

Raw data were obtained from an institution of higher learning in Johannesburg, South Africa. Four years of institutional data for undergraduate students from 2018 to 2021 enrolled for SP&A degree programmes were extracted from the university data warehouse. Variables were extracted that are typically cited in the literature to influence students’ performance, for example, financial, academic and demographic characteristics (Alyahyan & Düştegör, 2020). Table 1 provides the definition of the attributes. The variable in Table 1 includes the quantile ranking and different funding types. The quantile ranking system was implemented by the South African government to assist in redressing the past financial inequalities regarding educational funding in South Africa. All South African public schools are placed into five categories, called quintiles, which are used for the application of financial resources. Quintile 1 is the ‘poorest’ quintile whilst quintile 5 is the ‘least poor’. The quintile rankings are determined nationally according to the poverty of the community around the school and certain infrastructure factors (Van Dyk & White, 2019). A student may be self-funded or sponsored. Self-funded students may be paying their own school fees, or their guardians may be paying the fees. Students may be sponsored externally, for example, by a company or organisation. A student may also be sponsored internally, for example, through a staff bursary, faculty bursary or merit award that is sourced via the university’s financial aid and scholarship office.

**TABLE 1:** Attributes extracted from students’ records.

Attribute	Definition
Academic courses	The course(s) registered by the students, for example, SPPA1003A, with the medium of instructions for all courses being English
Programme	Whether a student is registered for Speech–Language Pathology or Audiology
University residence	Whether the student resides in the University residence or not
Funding (Funding types)	The source of financial support to cover a student’s tuition fees and living expenses.
Gender	Female, male or unspecified
Clubs or societies	Extramural activities that a student may take whilst on campus, for example, sports clubs or religious societies
Marital status	Whether the student is married, divorced, single
Age	The age of a student at the time of enrolment
Graduation	Indicates whether the student has graduated or not – in this or in any other programme
High school quintile	The government ranking of the high school that a student attended (an extended definition has been provided elsewhere)
Nationality	The citizenship of a student, for example, South African, Kenyan, etc.
Rural or urban	Whether the student’s home is located in an area that is classified as rural or urban
Year of study	Refers to the student’s current year of study
English	Students who speak English as their first language
Non-English	Students who do not speak English as their first language

### Data analysis

The data extracted from the university data warehouse had already undergone a thorough extraction, transformation and loading (ETL) process. The data were loaded onto Jupyter Notebook in Python, and the data cleaning process was conducted by deleting unusable columns of data, for example, incomplete columns of data sets from the biographical questionnaire. The extracted data were converted into a single file, with the columns representing the features and the rows representing students’ records. The attributes extracted and their definitions are given in Table 1. The data consists of 5608 horizontal rows of data, also known as observations, of students’ performance for 4 years. The final course mark is known as the target feature, and it represents the feature that the researchers were interested in gaining a deeper understanding of, because it determines whether a student has passed or failed. A statistical approach to assess and compare learning algorithms by dividing data into segments (known as cross-validation) was used. A 10-fold cross-validation was run with a split of 80% and 20% randomly shuffled testing and training sets, respectively. The extracted data were analysed using K means clustering and random forest classification. The summarised data were presented in tables and graphs.

### Ethical considerations

This study involved the use of anonymised data to evaluate student performance. Depersonalised attributes or features (for instance, course marks) were extracted from the database. The study followed all ethical standards of studies without direct contact with human or animal subjects. Ethical clearance to conduct this study was obtained from the University of the Witwatersrand, Human Research Ethics Committee (Non-Medical) (reference number: HRECNMW22/01/11).

## Results and discussion

**FIGURE 4:**
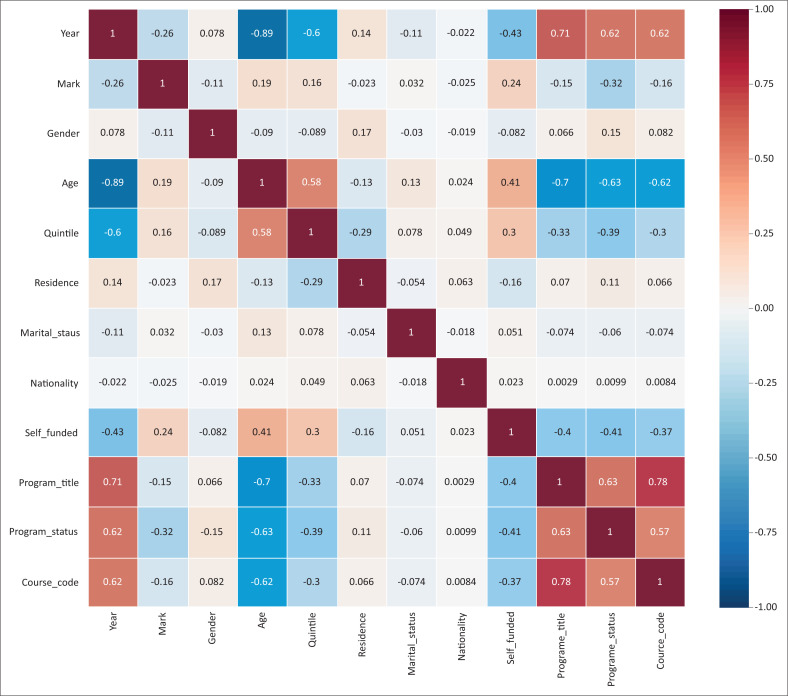
Correlation matrix indicating relationship between features.

In order to get general insights into the patterns and trends in the data set, a correlation matrix showing relationships between features in the data sets was drawn, as shown in Figure 4. There is a need to understand the relationship between variables. Each cell in Figure 4 shows the correlation between two specific variables. Features with a correlation number above 0.50 are considered to be strongly positive correlated, whilst features with negative numbers are weakly negative correlated, and numbers that are less than 0.006 are considered not to be correlated at all. The patterns observed on the correlation matrix indicated a correlation between students’ funding type and their final mark. There is also a positive correlation between self-funded students and the quintile. Age was also observed to have a positive correlation with the final mark. Therefore, from the correlation matrix, the students’ funding type, the courses registered by a student and age are viewed as important correlations. However, in the building of a student success or intervention model, features that introduce biases in the data set are not included.

**FIGURE 5:**
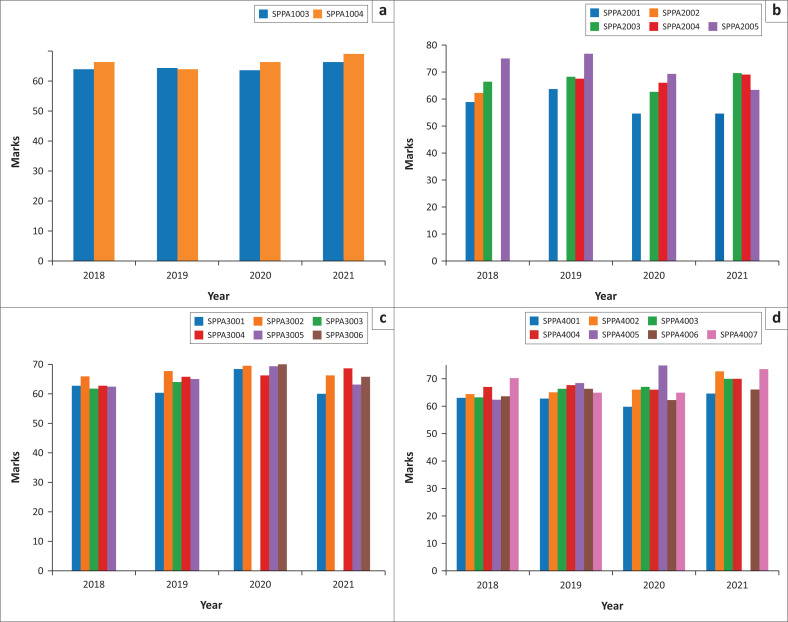
(a) First year students’ performance during face-to-face teaching and learning from 2018 to 2021, (b) course performance for second year, (c) students’ performance for third year and (d) students’ performance for fourth year.

Bar charts for overall students’ performance and performance per course were also drawn as shown in Figures 5a to 5d. Variations of average marks within the range of 5% are expected in student marks each year, because each cohort of students varies each year. The average performance of students can vary, even if the teaching practices remain the same. Despite the SP&A courses being taught by qualified and experienced staff each year, variations in the course marks are still expected, because the cohorts of students are different each year. The *p*-value and the confidence interval may be used to determine whether the average marks variation are statistically significant or not. A *p*-value of less than 0.05 was considered as the threshold of whether the average marks were statistically significant or not. Statistical significance does not imply practical significance; hence, further tests and interpretations are conducted for values deemed as statistically significant values. From 2018 to 2019, the average student performance was very similar for Speech and Hearing Science (SPPA1003A) and Speech Pathology and Audiology (SPPA1004A). There was then a slight increase in average marks for ERTL and hybrid learning (2020 and 2021) for SPPA1003 and SPPA1004, as seen in Figure 5a.

From Figure 5b, Audiology II (SPPA2001A) students’ average performance in 2020 and 2021 was lower compared with 2018 and 2019. There was also a slight drop in average performance for clinical practicals in Audiology (SPPA2005A) in 2020 and 2021. The SPPA2004 was introduced in 2019, and SPPA2002 was discontinued after 2018, hence the data are missing for those course codes.

In third year, students performed better in clinical practicals in Audiology (SPPA3004A) in 2021 compared with 2018 to 2020. In Speech–Language Pathology III (SPPA3005A), the highest performance was observed in 2020. The performance for SPPA3005A dropped back to its usual average in 2021 (Figure 5c). The SPPA 3003 was discontinued after 2019; hence, there is no data on the course after 2019.

Finally, in fourth year, observations show an improvement in average marks for SPPA4005 in 2020, as seen in Figure 5d. The SPPA4005 course was discontinued after 2020, hence the missing data in the year that follows. The SPPA4007 course shows similar performance in 2018 and 2021, whereas in 2019 and 2020 average performance is similar. The variations could be because of factors other than the shift to ERTL.

**FIGURE 6:**
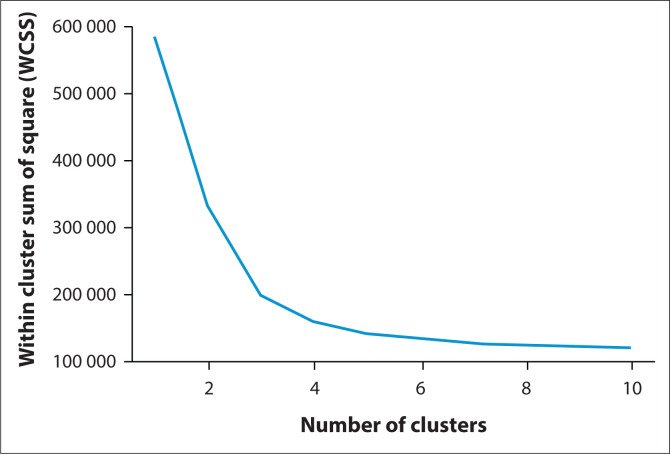
The elbow plot for optimal cluster selection.

The trends in the average marks plotted in the bar graphs (Figures 5a to 5d) indicate an increase in average marks in 2020 and 2021 for courses with practical components, except for one second-year course (SPPA2001) where there was a decrease in the average marks for a course with a practical component. Whilst the obtained general information is instrumental in providing a general view of the class performance over the years, it does not provide enough details to account for the reasons for the increase or drop in marks. Therefore, data are analysed further to obtain insights into students’ performance during face-to-face, ERTL and hybrid learning by clustering the students using K means. Clustering was conducted to determine the properties of the data set. The students were segmented into groups using K means. The elbow plot was used to determine the optimal cluster number that minimises the cost function, as shown in Figure 6. From the elbow plot, the optimal cluster number is three. The vertical axis is labelled as within cluster sum of square (WCSS) referring to WCSS, which is defined as the sum of squared distance between each point and the centroid in a cluster. When the WCSS is plotted with the *K*-value, the plot looks like an elbow. As the number of clusters increase, the WCSS value will start to decrease. The WCSS value is largest when *K* = 1, which is the optimal cluster selection.

**FIGURE 7:**
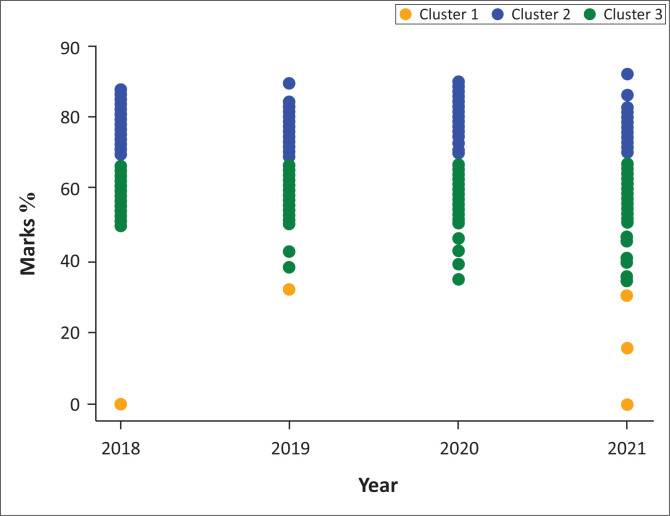
Third-year clusters from 2018 to 2021.

Each student in the entire data set was assigned to a cluster label. Figure 7 shows the clusters that were obtained using third-year students as an example.

Figure 7 shows three distinct priority classes in the students’ marks from 2018 to 2021. These priority classes are further broken down, as shown in Table 2.

**TABLE 2:** Students’ performance milestones.

Cut-off mark	Risk evaluation
69 to 100	Low risk
35 to 68	Medium risk
0 to 34	High risk

The change in students’ final course marks was the determining factor in classification into the level of priority. The classes in Table 2 can be used in the prediction of whether a given student will be able to complete the academic year or not, depending on their classification. Low-risk students are viewed to be generally performing well in their studies and they need minimal intervention; medium-risk students need intervention at a high level, which may be administered in a group setting. However, high-risk students are perceived to have various challenges with the courses and urgent intervention based on ‘one-on-one’ intervention may be required. At this point, the clusters still do not provide much information about the students’ attributes. In order to customise students’ interventions, it is important to understand the attributes that characterise each cluster. Information within the cluster is obtained using the random forest classifier.

**FIGURE 8:**
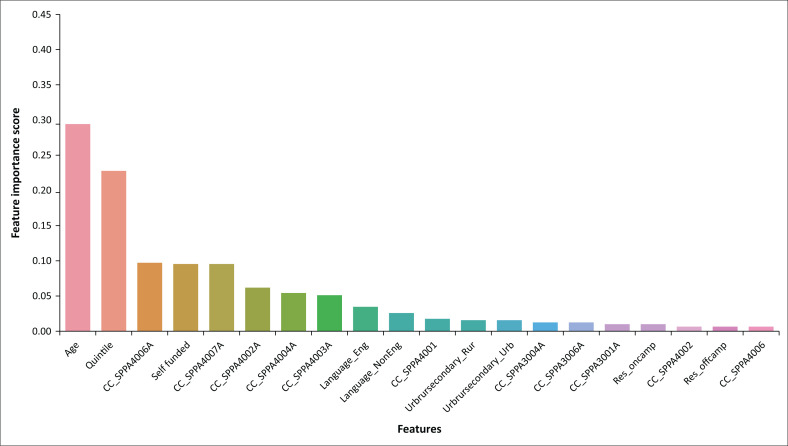
Fourth-year 2021 important attributes.

Figure 8 is an example of attribute extraction that was conducted using random forest. The main attributes that were found to affect students’ performance in the fourth year in 2021 are listed in order of priority starting with the age, quintile, SPPA4006 and funding. The first four attributes will be considered to be significant. The attributes are important because they provide the state of ‘academic health’ for a student. For example, if a student is self-funded and from a lower high school quintile (1, 2, 3), the department of SP&A will need to closely monitor the performance of this student, because the student is likely not to perform well. One of the courses that can be used to monitor students’ performance in fourth year would be the observed performance in SPPA4006A. The students’ performance in SPPA4006 strongly influences the performance in the other courses and the performance in their entire fourth year.

Classification of the data using random forest results is the ability to recognise the hidden patterns and trends in the data set, as shown in Table 3. The attributes vary depending on the year of study.

**TABLE 3:** Attributes in order of importance.

Year of study	2018	2019	2020	2021
1st year	Age	Age	Age	Age
	Quintile	Quintile	Quintile	Quintile
	ANAT1003A	LING1001A	SPPA1003	LING1001
	LING1001A	ANAT1003	SPPA1004A	Self-funding
	LING1003A	LING1003A	Self-funding	SPPA1003
	LANGUAGE– English	LANGUAGE Non English	MDLL105A	SPPA1004
	LANGUAGE- Non English	PSYC1009A	LING1001A	MDLL1015A
	SPPA1003	LANGUAGE – English	Language – Non English	MDLL1016A
	Urban/Rural Secondary	SPPA1003A	MDLL106A	LING1003A
2nd Year	Age	Age	Age	Age
	Quintile	Quintile	Quintile	Quintile
	LING2006	SPPA2003A	SPPA2001A	SPPA2001A
	SPPA2002	SPPA2004	SPPA2003	LING2006
	LANGUAGE – English	SPPA2001	LING2007	Self-Funded
			Self-Funded	
3rd Year	Age	Age	Age	Age
	Quintile	Quintile	Quintile	Quintile
	SPPA3005	LANGUAGE – Non English	Self-Funded	Self-funded
	PSYC3034	Language – English	PSYC3018A	LANGUAGE – Non English
	SPPA3003	PSYC3019	Psyc	SPPA3001A
4th Year	Age	Age	Age	Age
	Quintile	Quintile	Quintile	Quintile
	SPPA4007	SPPA4005	Self-Funded	SPPA4006A
	SPPA4006	SPPA4006	SPPA4005	Self-funded
	SPPA4005	LANGUAGE – English	LANGUAGE – English	SPPA4002A

The clusters were created using the attributes extracted and listed in Table 1. Random forest classifier was applied in order to understand the hidden information in each cluster for each year of study from 2018 to 2021. The observed patterns can be summarised as follows:

- Self-funded students from lower quintile high schools (1, 2 and 3) generally performed poorly from 2018 to 2021. Whereas, self-funded students from higher quintile schools (5, 6, 7) performed very well. This study does not consider whether the self-funded students paid their own tuition fees or if the tuition fees were paid by their guardians.

**FIGURE 9:**
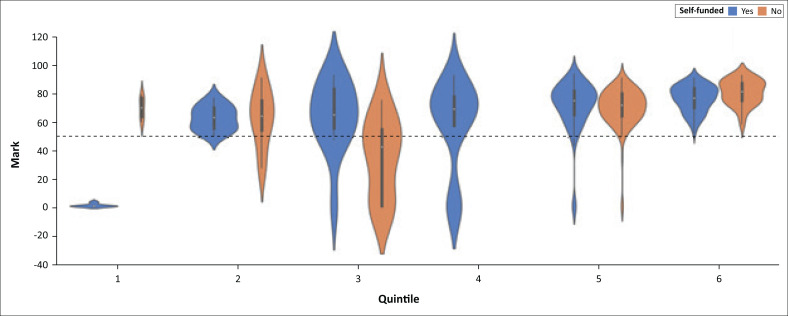
Relationship between self-funded students, the quintile and performance.

To understand and illustrate this finding further, Figure 9, is plotted to show the relationship between quintiles, funding and students’ performance.

- From 2018 to 2021, the age and high school quintile remained the priority factors for all the undergraduate students from first year to fourth year.
- Funding was observed to have a very low priority from 2018 to 2019 in determining a student’s performance; however, from 2020 to 2021, funding increased in priority and it influenced students’ performance.
- In 2018 and 2019, SPPA1003 had a lower priority; however, in 2020 and 2021, its priority increased and it became one of the key courses in first year that determined a student’s performance in first year.
- The SPPA2001 course influenced students’ performance in 2018, 2020 and 2021.
- In fourth year, SPPA4006A was a priority factor in 2018, 2019 and 2021.

These results indicate that one of the impacts of COVID-19 on SP&A students was having the financial health status of a student becoming one of the priority attributes that determined their success. Self-funded students from lower quintiles were affected more than self-funded students from higher quintiles. This is expected, because some of the effects of COVID-19 in South Africa were budget cuts and loss of income from different sectors. Institutions of higher learning were not an exception. It is not clearly understood from the data why SPPA1003 (Speech and Hearing Science), SPPA2001 (Audiology) and SPPA4006 (Research Report) became priority courses during the contact and blended learning. Careful deliberation of the content of these courses is required to ensure that the teaching and learning approaches adopted are responsive, relevant and responsible. The general insights and the important attributes from the clusters may be used in development of SP&A undergraduate students’ performance tracking system. A comparative analysis was also carried out using various classification techniques to establish the model that is suitable for the students’ data set. The results are shown in Table 4.

**TABLE 4:** Model performance.

Model	Average testing accuracy	Average training accuracy
Logistic regression	72.56	75.25
Support vector machines	86.10	99.89
Decision trees	91.32	99.90
Random forest classifier	91.86	99.84

The significance of the analytics in this study is that they can be used by various stakeholders to make predictions of student performance and be able to implement intervention strategies for students in a timely manner. The analytics indicate that each study approach has its own merits and demerits, and the different population groups of students are affected by the choice of one model of learning over the other. A hybrid type of learning would be more suitable because it is more flexible.

Although this study was prompted by the global negative effects of COVID-19 on teaching and learning (Maital & Barzani, 2020), current findings raise important implications going beyond COVID-19 if the hybrid model of teaching and learning remains part of standard practice. Within the SLPA programmes, where traditionally only face-to-face was the adopted model, the implications of these findings speak to areas requiring supportive measures for both access and success. The current findings were obtained using data from the SLPA programmes, however, the results can also be applied as evidence of South African colonialism- higher learning institutions, as described by Mgqwashu (2017) and Mpungose (2020).

If financial status in the form of self-funding is a critical factor in the performance of students during remote teaching and learning, as found in this study, it is important for institutions of higher learning to ensure that (1) students have funding; (2) they intensify their investment in digital technologies and digital literacy for both staff and students (García-Morales et al., 2021); (3) the Department of Higher Education and Training engages in and solidifies intersectoral collaboration with other relevant departments such as the Department of Communications to facilitate students’ access to Information and Communication Technology (ICT) resources and support for learning (Francis et al., 2020); (4) zero rating of all course material is negotiated as a standard nationally, without the current reported disconnect between sites that are zero rated and relevant ETRL relevant course material (Mhlanga, 2020; Tenet, 2021); (5) considerations are always made around power supply challenges such that students are not disadvantaged by load-shedding, which also affects connectivity that is beyond their control during remote learning (Azionya & Nhedzi, 2021; Laher et al., 2021; Oyedotun, 2020); (6) models of training forming part of remote teaching and learning as part of hybrid methodology such as telepractice, teletraining and use of simulations (Khoza-Shangase et al., 2021; Nagdee et al., in press) are carefully monitored for efficacy; and (7) psychosocial aspects such as gradual instead of abrupt change to remote teaching and learning (Thaba-Nkadimene, 2020), as well as provision of effective peer support and engagement platforms are considered (Chandra & Palvia, 2021).

## Limitations of the study

- It is assumed that the average performance can vary even if the teaching practices remain the same. Despite the SPPA courses being taught by qualified and experienced staff each year, variations in the course marks are still expected because the cohorts of students are different each year; therefore, definitive conclusions based on current data are limited, thus raising implications for future confirmatory research in this area.
- Some attributes (for instance, gender, disability, nationality and marital status) are analysed as attributes that affect student performance; however, in the future development of the ML model, these attributes are not included to avoid biases in the model.
- For self-funded students who have to work to pay their school fees, their specific performance based on this unique feature is not analysed in this study, which is thus an implication for future studies.
- The impact of race on student performance with respect to COVID-19 has deliberately been left out of this publication, even though it is well documented that attributes such as financial health remain racially linked in South Africa. This will be the focus in a future study.

## Recommendations and conclusion

According to the comprehensive analysis of the data sets, a novel approach in this field, both traditional methods of learning and online learning have their merits and demerits. Amalgamating the merits of both types of learning would result in a system that is easy for both students and lecturers or course instructors to navigate. Transitions towards blended or hybrid learning will require stakeholders to identify and merge the positive aspects from the online learning and face-to-face learning platforms. The COVID-19 pandemic has accelerated South Africa’s institutions of higher learning towards thinking about how to develop robust technological infrastructure that can be used alongside the traditional methods of teaching. The huge amount of data sets that institutions of higher learning have collected over the years can be used to analyse trends and patterns in students’ performance. This study has demonstrated how this can be done through ML, using SLPA as a case study. With this in mind, some of the questions that the SLPA programmes and institutions of higher learning generally will need to look at are:

- How can learning and teaching be improved to support student success?
- Which approach would be suitable to ensure students’ academic success?
- Which social, psychological and economic factors affect students, and what are the ways in which the students can work with the university to resolve the challenges?
- Which learning approaches are better suited for students, and is there a difference in these based on year of study?

The ML model can be used in implementation of an early warning and recommendation system that ensures that administrators provide early intervention to a student who is not performing well. However, it is important to note that features that would introduce biases in the data sets (for example, race, age, nationality, marital status and nationality) would not be included in the ML models. In future, the attributes that have been observed to have an influence on students’ performance will be used in the building of an early intervention model to help the course instructors and administrators in identifying students who are not coping with their workload and provide interventions. In the early intervention model, features that introduce biases in the data will not be included.
